# Knowledge, attitudes and practices of fresh Nile perch value chain handlers towards food safety requirements in Uganda

**DOI:** 10.1016/j.heliyon.2024.e31432

**Published:** 2024-05-20

**Authors:** Johnson A. Ssubi, Ivan M. Mukisa, Charles K. Muyanja

**Affiliations:** Department of Food Technology & Nutrition, School of Food Technology, Nutrition & Bioengineering, College of Agricultural and Environmental Science, P.O.Box 7062, Makerere University Kampala, Uganda

**Keywords:** Uganda, Nile perch value chain, Fish handlers, Food safety, Knowledge, Attitudes, And practices (KAP)

## Abstract

In the continuum from ‘farm to fork’, the proficiency of food handlers in terms of knowledge, attitudes, and practices (KAP) is essential for ensuring improved food safety outcomes. This study evaluated the KAP of fresh Nile perch fish handlers regarding food safety requirements within Uganda. A cross-sectional survey involving fish handlers (n = 466) engaged in handling fresh Nile perch and fisheries products in both local and export market chains. The study was conducted at 30 gazetted fish landing sites on Lake Victoria, 15 licensed fish export factories, and 9 local markets authorized to sell fisheries products. Data on demographic, knowledge, attitude, and practices was collected using structured questionnaire. Demographic data was analysed using frequencies and percentages. Ordinary least squares bivariate and multivariate regression analyses were performed to determine factors associated with knowledge, attitudes, and practices regarding food safety requirements. Fish handlers (49.8 %) demonstrated good knowledge with the majority (74.6 %) from the export market chain. Majority (81.3 %) of market stall handlers had poor knowledge of food safety requirements. Most (68.2 %) fresh Nile perch fish handlers had limited awareness of food-borne pathogens. Only 31.8 % had knowledge about *E. coli*, *Salmonella* spp, and *Staphylococcus* spp. Furthermore, over 63 % believed that consuming contaminated fish could transmit HIV, Covid-19, and Ebola. Male gender, higher income, and advanced education were positively correlated with fish handlers' knowledge of food safety requirements. Fish handlers (57 %) in the local market chain exhibited poor attitudes. Being a male fish handler was significantly (**β** = 3.43, 95 % CI: 1.65–5.21, P = 0.001) associated with positive attitudes compared to being female. Education at different levels was significantly (Primary (β = 3.19, 95 % CI: 1.05–5.33, P = 0.004); Secondary level (β = 5.883, 95 % CI: 3.52–8.23, P = 0.001); Tertiary (β = 6.09, 95 % CI: 3.03–9.15, P = 0.001)) associated with positive attitudes compared to no education.

Practices ensuring food safety were common in the factories and the export market. The study suggests the need for interventions that promote knowledge transfer and food safety culture, particularly in the local market chain.

## Highlights

• Food safety knowledge and attitudes vary amid export and local value chain workers.
• Food safety practices significantly differ along the fresh Nile perch value chain.
• Gender significantly influences food safety knowledge, attitudes and practices.
• Food safety regulatory monitoring varies between the export and local value chains.
• Knowledge transfer and behavioural change vital for improved food safety outcomes.

## 1 Introduction

Globally, the per capita consumption of fish rose from 9 kg in 1961 to 20.5 kg in 2018, reflecting a 15 % annual growth [1]. Accordingly, the per capita consumption is expected to rise to 21.5 kg by 2030 in the developed countries with a decline of 0.2 % in the less developed countries, especially in Sub-Saharan Africa. The Food and Agriculture Organisation further reported that fish consumption alone in 2017 contributed about 17 % of the world's population animal protein intake and 7 % of the total protein intakes [1,2].

In Uganda, the fisheries subsector, ranking third after gold and coffee, contributes over 5 % to the country's GDP [3,4]. Despite this, there is declining (from 19.1 kg in 1977 to the current 14.3 kg) per capita fresh water fish consumption in Uganda due to a reduction in the fish stocks in Lake Victoria [5], and increasing exports where Nile perch contributes about 90 % of the total fish exports [6].

Although some of the water bodies are at risk of contamination by chemicals and biological agents which negatively impact the food safety of fish [7], it is believed that fish from open fresh waters is safe if handled and stored properly. To ensure food safety along the fish value chain, food trade regulations, as stipulated by the World Trade Organisation (WTO), require member states to adhere to sanitary and phytosanitary measures based on risk assessment [8].

In the late 1990s, Uganda suffered fish export bans by the European Union (EU) due to concerns of contamination in fish exports, which prompted strengthening of the regulatory environment to meet international standards. Strict regulatory requirements and controls were imposed on all fish exports from East Africa to cover aspects of good manufacturing practices (GMP), application of HACCP and international standard requirements such as ISO 9001:2000 for the sustainable production of high quality fisheries products [9]. These measures led to improvement in compliance to standards and other food safety requirements, at least for the export fisheries products. However, various authors [[10], [11], [12]] expressed public concern regarding the safety of locally sold fisheries products, with Amos et al. [13], emphasizing the critical role of fish handlers' behaviours and practices in food safety.

Several studies have highlighted unsafe water for cleaning and processing, poor production processes, inadequate storage, food handling practices and cross contamination of food as some of the underlying conditions for contamination of food with microbiological hazards [14,15]. Furthermore Christiana Cudjoe et al. [16] and da Vitória et al. [17], highlighted the positive association between improper food handling, poor hygiene, education levels, and foodborne illnesses. Bedane et al. [18] emphasized the importance of personal hygiene awareness (particularly handwashing, among food handlers) in preventing contamination since food handlers are important carriers of food borne illnesses. While some authors have reported the level of food safety knowledge to be related to the food handlers attitudes and practices [19], other researchers have reported attitude as a fundamental factor that could influence food safety behaviours and practices [20,21]. Subsequently, interventions based on knowledge, attitudes and practices (KAP), are more frequently being used to identify and address food safety concerns at various stages of the food value chain [22].

Despite the significance of proper food handling and knowledge transfer in ensuring food safety [23,24], the food safety knowledge, attitudes, and practices (KAP) of fish handlers along the fresh Nile perch value chain in Uganda remains unknown. This study set out to fill this knowledge gap by assessing the KAP of fish handlers along the fresh Nile perch value chain in Uganda, with the view of understanding factors that influence food safety. This information is critical for guiding policy and intervention strategies to promote food safety in the fresh Nile perch value chain in Uganda, particularly the safety of locally sold fisheries products.

## 2 Materials and methods

### 2.1 Study design and location

This cross-sectional study was conducted between September and December 2022, at different facilities handling fresh Nile perch and its products for placing on the market in Uganda. Visits were made to all the registered landing sites around Lake Victoria, local market stalls around the Lake Victoria basin and fish processing factories in Uganda. All the fish handling facilities that were operational during the study period as shown in tables ST1, ST2, and ST3, were included in this study. The study was conducted at 30 landing sites (15 of which were handling fish for export (Table ST1) with the remaining 15 (located adjacent to the export fish handling areas) handling fish going to the local market). In addition, 9 market stalls and 15 fish processing factories were included, that were operational at the time of the study.

### 2.2 Determination of number of fish handlers for the food safety KAP study

The total number of fish handlers (n) who participated in the study was obtained using Yamane equation [25];

$$n=N/\left(1+N\left(e^{2}\right)\right)$$

Where n is the sample size, N is the population size, and e is the level of precision equivalent to 5 % with a 95 % confidence interval. The overall population of handlers in the Nile perch value chain around the Lake Victoria basin including factories and markets was estimated by the Department of Fisheries Resources at 30,878 people. Using the above Yamane equation, the overall sample size (n) was calculated as 395.

The number of fish handlers per fish handling facility (x) was then calculated as a proportion of the overall sample size (n) based on the population (y) that facility contributed to the overall population (N) and then increased by 10 % to cover non-response.

x=(y/N)(n)+(10/100)*((y/N)(n))

The 10 % correction to cover for non-response per facility, overall, increased the final total sample size to 466 fish handlers. The number of fish handlers per facility is indicated in Tables ST1, ST2, and ST3. Fish handlers were selected based on their availability and willingness to participate.

### 2.3 Data collection

Primary data were collected using a self-administered, pre-coded questionnaire prepared in English. The questionnaire was piloted on 50 fish handlers who were not included in the final survey. The necessary modifications were made on a number of questions to improve clarity, before the questionnaire took its final form. The questionnaire aimed to gather data that provides an understanding about the extent of knowledge, attitudes, and practices on food safety requirements along the fresh Nile perch value chain. The questionnaire had four sections (I-IV): Section **I**- demographic information; **section II-** knowledge on: sanitation and health, good personnel hygiene, fish spoilage, - fish handling, processing and preservation, food hazards, contamination, and cross contamination; **section III-** attitudes; **section IV**- practices.

### 2.4 Data analysis

Socio-demographic characteristics were presented using frequencies and percentages. Cross-tabulations and the ꭓ^2^ test were used to determine differences in knowledge, attitudes, and practices between local and export market actors, and across value chain segments. Principal Component Analysis (PCA) was used to summarize knowledge and attitude attributes of food safety requirements. Binary variables were created for each of the questions in each section of the questionnaire. Specifically, for the knowledge section, responses were scored as either “good” or “poor” knowledge (i.e. an incorrect response was scored as “poor” whereas a correct response was scored as “good”). For the attitude section, the responses were redefined into two groups. i.e “strongly agree” and “agree” responses were categorised as “good attitude” whereas “no idea”, ‘disagree” and “strongly disagree” responses were categorised as “poor attitude”. Further, responses to knowledge and attitude questions were combined and a cumulative score on a continuous scale was generated for each respondent. With this score, ordinary least squares regression method was used to identify factors influencing knowledge and attitudes of fish handlers. Starting with bivariate analysis, variables reporting P-values ≤0.2 were incorporated into the multivariate model. Statistically significant associations were tested for at the 5 % level of significance. The analysis was conducted using STATA V15 [26].

## 3 Results and discussion

### 3.1 Socio-demographic characteristics of fish handlers

The socio-demographic characteristics of the fish handlers are presented in table 1.

The results showed that out of the 466 fish handlers that participated in this study, the majority (86.5 %) were males and within an age range of 20–40 years. The highest level of education attained by majority of the fish handlers was primary level (42.3 %) and 58.6 % of the fish handlers had received some form of food safety training. The majority of the fish handlers were workers (72.5 %), mostly handlers (44.9 %), majority (55.6 %) earning less than 300,000 Uganda shillings (approximately $75 USD) per month.

### 3.2 Fish handlers’ knowledge about food safety requirements

From Tables 2a and 2b, it can be noted that 76.0 % of the fish handlers knew that washing hands using only water does not reduce the risk of microbial contamination of fish. This finding was in agreement with studies by Akabanda et al. [27] and Hashanuzzaman et al. [22]. These authors reported that 72.8 % and 100 % of the food handlers respectively, knew that washing hands and utensils with detergent or soap was important to prevent contamination. This could be explained by the fact that more than 58 % and 91 % of the food handlers in the current study had food safety training and formal education, respectively. This was neither significantly different between the different market chain sectors (P = 0.226) nor among the value chain value segments (P = 0.242).

The results from this study showed that 395 (84.8 %) of the fish handlers reported that using gloves when handling fish reduces the risk of food contamination. Similar findings were reported in Ghana [27]. However, the score in this study was lower than the 100 % reported in Nigeria [28]. The results obtained in this study were contradictory to those reported in Bangladesh where only 32 % of the food handlers recognised the importance of physically handling food with gloves [22]. This knowledge about wearing gloves before touching fish could be attributed to the fact that more than 58 % of the handlers had received some form of food safety training. Whereas this was significantly (P < 0.001) different between the market chain sectors, no significant (P = 0.484) differences were observed among the value chain segments. According to Todd [15], there was a difference in the management of businesses, with the export market chain being more compliant to the food safety guidelines for fear of rejection of the exported fish, whereas the local market dealers mostly operated in poorly implemented food safety management systems. This difference in management may be an explanation for the significant difference in the level of knowledge of handlers between the export and local market chains.

Fish handlers (68.2 %) had no knowledge about *E.coli, Salmonella* spp, and *Staphylococcus* spp as food-borne pathogens. The findings obtained in this study were in agreement with those reported in Ghana where more than 76 % did not know that *Salmonella* is a food borne pathogen [27]. Another study in Nigeria in 2019 reported that 67 %–78 % of the food handlers also did not know salmonella as a food borne pathogen [28]. On the contrary, the results obtained in this study differed from those reported in Saudi Arabia where more than 73 % of the fish handlers were knowledgeable about the foodborne pathogens [29]. There was significant (P < 0.001) difference between the market chain sectors and among the value chain segments (P < 0.001) in regard to knowledge about foodborne pathogens.

The majority (63.7 %) of the fish handlers exhibited poor knowledge about food borne diseases, as evidenced by wrongly stating that diseases like HIV, COVID-19 and Ebola could be transmitted through consumption of contaminated fish. This finding points to an apparent need for the regulatory authorities, such as the Ministry of Health, to undertake public health awareness campaigns among the population in Uganda. Sensitization drives on food borne illnesses and their etiology would greatly improve food safety outcomes within the fresh Nile perch value chain in Uganda.

### 3.3 Fish handlers’ attitudes towards food safety requirements

**Fig. 1:**
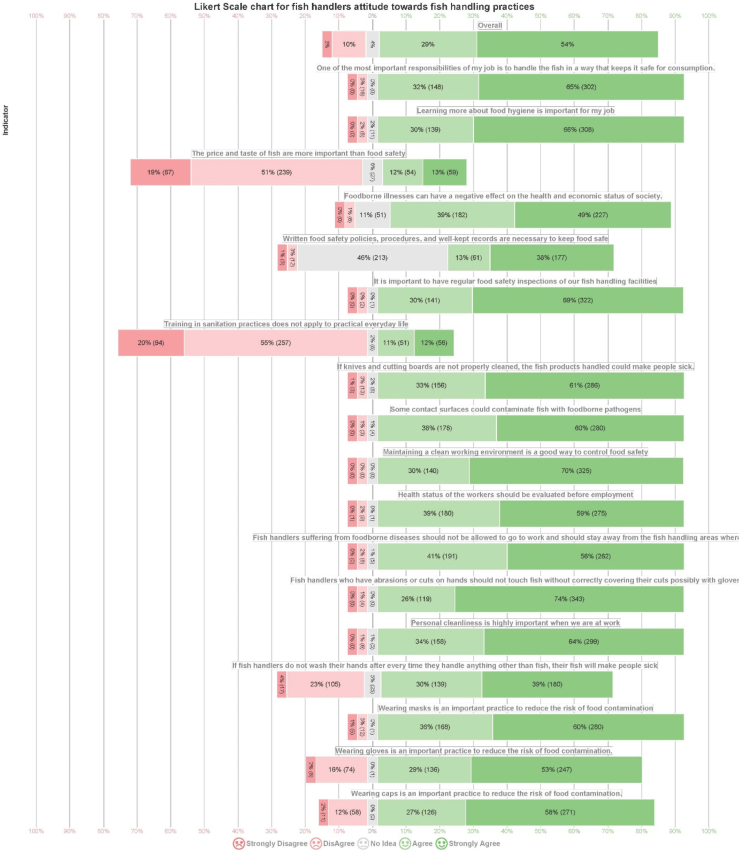
Overall scores for Fish handlers' attitude towards food safety requirements within the fresh Nile perch value chain in Uganda

Fig. 1, Tables 3a and 3b, show the attitudes of fish handlers along the fresh Nile perch value chain towards food safety requirements, and how these attitudes differ by market chain sectors and segments of the value chain. The occurrence of foodborne illnesses can greatly be reduced or prevented if the food handlers can develop positive attitudes towards food safety guidelines or standards [27]. From Fig. 1, the majority (64.8 %) of the fish handlers strongly agreed that one of their most important responsibilities was to handle the fish in a way that keeps it safe for consumption. This was in line with a study done in Ethiopia among the fruits and vegetables value chain handlers [30]. However in this study, the attitude score is much higher than in a similar study done in Egypt where only 43.8 % strongly agreed that it was their mandate to keep the fish safe for consumption [31]. There was statistical significance between the market chain sectors (P < 0.001) (table 3a) and among the value chain segments (P < 0.001) (table 3b). Further, a majority (51.3 %) of the fish handlers disagreed or strongly disagreed that the price and taste of fish are more important than food safety. This was significantly highest in those that produce for export market compared to the local market (P = 0.016) and significantly highest in those at the factory compared to landing sites and compared to market stalls (P < 0.001). Overall, 69.1 % of the fish handlers strongly agreed that it is important to have regular food safety inspections of fish handling facilities. As seen in Tables 3a and 3b respectively, this attitude was significantly higher in those producing for the export market (P = 0.002) and in those at the landing site (P = 0.020).

More than 61 % of the fish handlers indicated that improper cleaning of knives and cutting boards where the fish products are handled could lead to contamination hence posing a health risk to consumers. This is a positive attitude reported by the food handlers and is in agreement with the results of other authors [27,32,33].

Findings also indicated that more than 62 % of the fish handlers strongly agree that personal cleanliness is highly important when at work in a fish handling facility. This is crucial in ensuring food safety and it is consistent with the findings reported in Saudi Arabia [29]. However the results in this study were lower than in Nigeria where a 100 % positive attitude was reported [28]. All the above positive attitudes towards food safety could be attributed to the fact that most of the fish handlers (61 %, 58.6 % and 91 %) had more than six years’ experience in business, with training in food safety and formal education respectively.

With respect to food safety policies, only 38 % of the fish handlers strongly agreed that written food safety policies, procedures, and well-kept records are necessary to keep food safe, implying that the remaining 62 % had a relatively negative attitude. This negative attitude would lead to poor practices that negatively influence food safety outcomes within the fresh Nile perch value chain. It is therefore prudent that government regulatory authorities and policy makers such as the Department of Fisheries Resources, and the Uganda National Bureau of Standards engage with fish handlers along the fresh Nile perch value chain to sensitize them on the basic food safety requirements to ensure improved food safety outcomes.

More than half of the fish handlers strongly agreed that wearing gloves is an important practice to reduce the risk of food contamination. This is a positive attitude that needs to be encouraged since the hands of food handlers are regarded as one of the important drivers of pathogens from bodies and other environmental surfaces to foods [34].

### 3.4 Fish handlers’ food safety practices

Tables 4a and 4b show the practices of fish handlers regarding food safety requirements by the different market chain sectors and value chain segments. Overall, 63.1 % of the fish handlers reported municipal water as the source of water for cleaning. This practice was significantly (P < 0.001) higher in those producing for export market (90.9 %) compared to those producing for the local market (30.4 %). The use of municipal water as a source for cleaning water, was significantly (P < 0.001) higher in those at the market stalls (96.9 %) compared to those at the factory (84.6 %) and at the landing sites (56.4 %). Overall, 43.6 % of the fish handlers reported that they kept fish for as long as 12–24 h. This practice of keeping fish for long hours, was significantly (P < 0.001) higher in those producing for the local market (51.9 %) compared to those producing for the export market (36.5 %); and significantly (P < 0.001) higher in those at the landing site (46.9 %) compared to those at the market stalls (28.1 %) and those at the factory (32.3 %).

Results in table 4b further show that food safety practices like washing hands with soap, water and sanitizer were more practised (93.8 %) at the factories than in other places. The same trend was observed for washing hands before touching the fish. Majority (85.7 %) of the fish handlers adhering to this good practice were identified in the export value chain and at the factory (87.7 %) as compared to their counterparts at the landing sites (56 %) and market stalls (56.3 %), table 4a. These findings were in agreement with those reported by other authors in Egypt [31] and in Nigeria [28]. This could be because the factories predominantly process for the export market. Whereas the factories formally target to recruit “elite” workers who are able to easily comprehend and implement food safety guidelines, recruitment at the market stalls and landing sites is purely informal and guided by the demand for casual labourers. The results in this study agree with those reported in Nigeria where a similar trend in the practices towards food safety was reported [32].

Fish handlers across all segments of both the export and local value chains, scored above 90 % about performing quality checks on the fish they handle per batch. This is a key practice in fostering food safety and ensuring that consumers are protected against foodborne illnesses. However, regulatory monitoring of facilities was more within the export value chain (at 57.8 % quarterly and 33.5 % monthly inspections) than in the local market value chain (at 44.4 % quarterly and 29.9 % monthly inspections). Further still, the regulatory monitoring was reported to be most vigorous at factory level (78.1 % quarterly and 21.9 % monthly inspections), as compared to market stalls (53.1 % quarterly and 28.1 % monthly inspections). The landing sites were reported to be at (46.9 % quarterly and 33.9 % monthly inspections). This variation in regulatory monitoring may be responsible for some of the differences in the knowledge, attitudes and other practices of value chain actors observed in this study.

Table 5 summarises the scores of the different indicators used to assess fish handlers’ knowledge and attitude towards food safety requirements. It was observed that overall, 49.8 % of the fish handlers had good knowledge of the food safety requirements, of which 74.6 % of the actors in the export value chain had good knowledge. Only 20.6 % of the actors in the local market chain had good knowledge of food safety requirements. There was statistical significance with Chi-square P < 0.001 in the level of knowledge. The overall level of knowledge observed in this study, was lower than the level of knowledge (77.8 %) reported in a similar study in Nigeria [32] but higher than the level of knowledge among fish handlers in Egypt [31].

The majority (81.3 %) of the fish handlers at the market stalls had poor knowledge of food safety requirements while the majority (89.2 %) of the handlers at the factory had good knowledge of food safety requirements. These differences in level of knowledge were statistically significant (P < 0.001). At landing sites, the level of knowledge was almost evenly distributed (good knowledge 45.5 % and poor knowledge 54.5 %), most probably because the landing sites were equally distributed between the export and local market value chains.

Overall, a smaller proportion of the fish handlers (27.5 %) had a good attitude towards food safety requirements. This percentage was much lower than that reported in a similar study in Egypt [35]. These authors reported that 98 % of fish handlers had a good attitude towards food safety requirements. The percentage of handlers with a good attitude in this current study was also lower that what was reported in another Egyptian study which found 61.2 % of the fish handlers having good attitudes towards food safety requirements [31]. The difference observed between the current study and those done elsewhere could be due to the differences in the study population (differing levels of education and development), time and study setting.

The attitude towards food safety requirements within the fresh Nile perch value chain in Uganda significantly (Chi-square P < 0.001) varied between the export market chain and the local market chain. The fish handlers in the export value chain had either a good attitude (42.5 %) or a fair attitude (44.5 %) towards food safety requirements, while the counterpart fish handlers in the local market value chain had either a poor attitude (57.5 %) or a fair attitude (32.5 %) towards food safety requirements. The attitude towards food safety requirements within the fresh Nile perch value chain in Uganda, also significantly (Chi-square P < 0.001) varied between factories, landing sites, and market stalls. It was noted that a smaller proportion of the fish handlers at the market stalls (12.5 %) and at landing sites (24.9 %) had a good attitude towards food safety requirements, compared to the relatively big proportion (49.2 %) of the handlers at the factory who had a good attitude towards food safety requirements.

The differences observed in the attitudes towards food safety requirements among the fish handlers, could be a contributing factor to differences in the microbial food safety of the fisheries products within the different market chains and value chain segments. It is important that the regulatory authorities in the fresh Nile perch value chain in Uganda target resources towards mind-set change as one of the ways to improve food safety within the fisheries sub sector.

### 3.5 The factors associated with level of knowledge of food safety requirements among fish handlers within the fresh Nile perch value chain in Uganda

Table 6 shows the bivariate and multivariate association of market chain and knowledge of food safety requirements. In the bivariate analysis, the knowledge score of the fish handlers within the local market chain was significantly reduced by 2.63 units (95 % CI: −3.02, −2.24) compared to those within the export market chain. After adjusting for socio-demographic characteristics of the fish handlers, the fish handlers within the local market chain were 1.87 times less likely to be more knowledgeable about fish safety than those within the export market chain (β = −1.87, 95 % CI: −2.35, −1.39, P = 0.001).

This study noted that the sex of the fish handlers was positively associated with knowledge score of the food safety practices (β = 0.81; 95 % CI:0.26–1.36, P = 0.004). Males were 0.81 times more likely to be knowledgeable than females. These results were contrary to those reported in Nigeria where no association between sex and the level of knowledge about food safety was observed [32].

Earning between 300,000 and 600,000 Uganda Shillings (approximately USD 75–150 $) a month (a moderate income) was also positively associated with food safety knowledge (β = 0.72; 95 % CI: 0.26–1.17, P = 0.002). Those earning between 300,000 and 600,000 were 0.72 times more likely to be knowledgeable than their counterparts that earned less than 300,000Shillings (a low income). The finding of this study were consistent with those of a study done on food safety attitude and associated factors among mothers of under 5 children, in Debarq Town [33].

From the results, the education level of the fish handlers was significantly positively associated with the level of knowledge about food safety (β = 1.64, 95 % CI 0.70–2.58, P = 0.001). This was in line with studies conducted in, Jordan [36], Ethiopia [30] and Egypt [35]. These authors reported that the education level of the food handlers was positively associated with the level of food safety knowledge, attitudes and practices [30,35,36].

Previous training of the fish handlers about food safety was positively associated with the level of knowledge about food safety at the time of this study (β = 0.97, 95 % CI 0.53–1.40, p = 0.001). This finding was consistent with other studies in low-income and middle-income countries where handlers that had previous training in food safety were 4.64 times more likely to have good knowledge, attitudes and practices about food safety [35,37,38].

On the other hand, the results indicated that some fish handlers’ socio-demographic characteristics had a negative association with the food safety knowledge score. For instance, fish handlers with 11–15 years of business experience were 0.85 times less knowledgeable about food safety than those with 5 years or less of business experience. This finding contradicted with findings of the study done in Nigeria where having been in business for more than 4 years was positively associated with good food safety knowledge [32].

### 3.6 The factors associated with level of attitude of fish handlers towards food safety requirements within the fresh Nile perch value chain in Uganda

Table 7 shows association of market chain sectors and attitude score towards the fish safety requirements. In the bivariate analysis, it can be deduced that the attitude of fish handlers within the local market chain significantly reduced by 7.79 units (95 % CI: −8.96, −6.62) compared to the attitude of fish handlers within the export market chain. This would imply that the fish handlers within the local market chain are 7.79 times less likely to have positive attitudes towards food safety requirements than the fish handlers in the export market chain.

After adjusting for the socio demographic characteristics in the multivariate analysis, this significantly reduced by 5.80 units (95 % CI: 7.36, −4.24) compared to the attitude of the fish handlers within the export market chain. Fish handlers within the local market chain were 5.8 times less likely to have positive attitudes towards food safety requirements compared to fish handlers within the export market chain.

This current study also noted that sex and level of education were significantly associated with attitudes towards food safety requirements. The results obtained in this study are similar to those reported by other authors in other countries for various value chains, Ethiopia [39], Egypt [31], and Bangladesh [38]. In this study it was observed, that being male was significantly associated with positive attitudes towards food safety requirements (**β** = 3.43, 95 % CI: 1.65–5.21, P = 0.001), compared to being female. This study also revealed that having primary level of education (**β** = 3.19, 95 % CI: 1.05–5.33, P = 0.004), secondary level (**β** = 5.883, 95 % CI: 3.52–8.23, P = 0.001) and tertiary level (**β** = 6.09, 95 % CI: 3.03–9.15, P = 0.001) were significantly associated with positive attitudes towards food safety requirements compared to no formal education.

### 3.7 Limitations to the study

The data in this study was self-reported which is a limitation to our study as the fish handlers may have been subject to social desirability bias and/or erroneous reporting due to recall failure. Furthermore, the study was of a cross-sectional design, which does not allow causal relationships to be established. Further research involving direct observations of food safety practices over longer periods is needed to derive more accurate conclusions on food safety knowledge, attitudes and practices within the fresh Nile perch value chain in Uganda. A “Cause - effect analysis” on the variation in the regulatory monitoring practices between the export and local market chains should be undertaken to further provide scientific guidance on the most appropriate interventions for improved food safety outcomes within the fresh Nile perch value chain.

## 4 Conclusions

In order to develop policies and programs that ensure improved food safety outcomes, it is prudent to understand the food handlers' level of KAP regarding food safety requirements. This study was conducted to evaluate the KAP of fresh Nile perch fish handlers regarding food safety requirements within Uganda. The study concluded that fish handlers within the fresh Nile perch value chain in Uganda had some minimum food safety knowledge (49.8 %), with above average positive attitudes towards food safety requirements. The fish handlers within the export market chain were particularly more knowledgeable than their counterparts in the local market chain. This study further concluded that male fish handlers were more knowledgeable about food safety with more likelihood of positive attitudes towards food safety requirements that female fish handlers. Thus, at operational level, knowledge transfer interventions targeting female fish handlers within the local market chain are still required in order to improve food safety outcomes within the fresh Nile perch value chain in Uganda. At policy level, interventions should aim at improving the fish handlers’ food safety culture through continuous food safety training, awareness creation and motivation of all fish handlers particularly the women, low-income earners, and the less educated within the local market chain.

## Ethical statement

The author(s) declare that the study did not pose any health or psychological risks to the fish handlers, and that all fish handlers provided informed written consent prior to being interviewed. Permission to conduct the study was obtained from the Fisheries Officers at the public fish handling facilities, and the Managers at the privately owned fish handling facilities. Fish handlers were selected basing on their interest to participate, and were above 18 years (consenting adults as provided for by the Constitution of the Republic of Uganda). The cross-sectional study collected descriptive data, where responses obtained had no personal, social or political consequences.

## Data availability

The datasets generated during and/or analysed during the current study are not publicly available, but can be accessed from the corresponding author on request.

## Funding statement

This research did not receive any specific grant from funding agencies in the public, commercial, or not-for-profit sectors.

## CRediT authorship contribution statement

**Johnson A. Ssubi:** Writing – review & editing, Writing – original draft, Resources, Project administration, Methodology, Investigation, Funding acquisition, Formal analysis, Data curation, Conceptualization. **Ivan M. Mukisa:** Writing – review & editing, Supervision, Methodology, Investigation. **Charles K. Muyanja:** Writing – review & editing, Supervision, Methodology, Investigation.

## Declaration of competing interest

The authors declare that they have no known competing financial interests or personal relationships that could have appeared to influence the work reported in this paper.

## Figures and Tables

**Table 1:** Socio-demographic characteristics of fish handlers within the fresh Nile perch value chain in Uganda

Characteristics	Frequency (N = 466)	Proportion (%)
Gender of respondent		
Female	63	13.5
Male	403	86.5
**Age category**		
18–20yrs	9	1.9
20–30yrs	189	40.6
31–40yrs	185	39.7
Above 40yrs	83	17.8
**Highest Education level attained**		
No formal education	41	8.8
Primary	197	42.3
Secondary	153	32.8
Tertiary	75	16.1
**Average monthly income (Uganda shillings)**^a^		
<300,000	259	55.6
300,000–600,000	147	31.5
600,001–900,000	40	8.6
>900,000	20	4.3
**Status in the value chain**		
Fisherman	23	4.9
Transporter	30	6.5
Handler	209	44.9
Supervisor/Manager	87	18.7
Trader	116	24.9
**Years of business experience**		
5yrs or less	174	37.3
6–10yrs	166	35.6
11–15yrs	49	10.5
>15yrs	77	16.5
**Role of Respondent**		
Worker	338	72.5
Owner	128	27.5
**Had any Food Safety training**		
No	193	41.4
Yes	273	58.6

a 1 Uganda Shilling is approximately equivalent to 0.00026 USD.

**Table 2a:** Food safety knowledge among the fish handlers in the fresh Nile perch Market value chain in Uganda

Characteristic	Overall	Market chain of interest		
		Export	Local	
	Freq. n = 466(percentage)	Freq. n = 252(acol%)	Freq. n = 214(col%)	X^2^ P-value
Water alone does not reduce microbial contamination on hands	354 (76.0)	197(78.2)	157(73.4)	0.226
Use of gloves reduces the risk of food contamination	395 (84.8)	235(93.3)	160(74.8)	<0.001
Eating from the workplace increases the risk of food contamination	306 (65.7)	220(87.3)	86(40.2)	<0.001
Proper cleaning of fish contact surfaces reduces the risk of food contamination	459 (98.5)	248(98.4)	211(98.6)	0.87
Washing equipment without soap leaves them free of contamination	405 (86.9)	217(86.1)	188(87.9)	0.579
All age groups are at equal risk for food poisoning	89 (19.1)	19(7.5)	70(32.7)	<0.001
HIV, COVID-19 and Ebola can be transmitted by eating contaminated fish	297 (63.7)	210(83.3)	87(40.7)	<0.001
Diarrhoea, dysentery, typhoid, cholera may result from eating contaminated fish	380 (81.5)	205(81.3)	175(81.8)	0.906
*E.coli, Salmonella, Staphylococcus* are food borne pathogens	148 (31.8)	129(51.2)	19(8.9)	<0.001
Food borne pathogens naturally exist in the environment	392 (84.1)	230(91.3)	162(75.7)	<0.001
Some pathogens are waterborne and may contaminate fishery products	413 (88.6)	241(95.6)	172(80.4)	<0.001
Cross contamination is transfer of microorganisms from contaminated sources	274 (58.8)	184(73.0)	90(42.1)	<0.001
Freezing kills bacteria causing food-borne illnesses	158 (33.9)	103(40.9)	55(25.7)	0.001
Contaminated fish always changes colour, odour or taste	14 (3.0)	14(5.6)	0(0.0)	<0.001
Fresh fish products should be stored below 4^0^C	177 (38.0)	144(57.1)	33(15.4)	<0.001

a col% = Column percentage.

**Table 2b:** Food safety knowledge among the fish handlers in the fresh Nile perch Segment of the value chain in Uganda

Characteristic	Overall	Segment of the value chain			
		Landing site	Market stalls	Factory	
	Freq. n = 466(percentage)	Freq. n = 369 (∗col%)	Freq. n = 32(col%)	Freq. n = 65(col%)	X^2^ P-value
Water alone does not reduce microbial contamination on hands	354 (76.0)	279(75.6)	28(87.5)	47(72.3)	0.242
Use of gloves reduces the risk of food contamination	395 (84.8)	316(85.6)	25(78.1)	54(83.1)	0.484
Eating from the workplace increases the risk of food contamination	306 (65.7)	231(62.6)	14(43.8)	61(93.8)	<0.001
Proper cleaning of fish contact surfaces reduces the risk of food contamination	459 (98.5)	364(98.6)	31(96.9)	64(98.5)	0.732
Washing equipment without soap leaves them free of contamination	405 (86.9)	320(86.7)	29(90.6)	56(86.2)	0.806
All age groups are at equal risk for food poisoning	89 (19.1)	74(20.1)	5(15.6)	10(15.4)	0.592
HIV, COVID-19 and Ebola can be transmitted by eating contaminated fish	297 (63.7)	213(57.7)	24(75.0)	60(92.3)	<0.001
Diarrhoea, dysentery, typhoid, cholera may result from eating contaminated fish	380 (81.5)	296(80.2)	31(96.9)	53(81.5)	0.066
*E.coli, Salmonella, Staphylococcus* are food borne pathogens	148 (31.8)	93(25.2)	3(9.4)	52(80.0)	<0.001
Food borne pathogens naturally exist in the environment	392 (84.1)	310(84.0)	24(75.0)	58(89.2)	0.195
Some pathogens are waterborne and may contaminate fishery products	413 (88.6)	321(87.0)	30(93.8)	62(95.4)	0.093
Cross contamination is transfer of microorganisms from contaminated sources	274 (58.8)	200(54.2)	14(43.8)	60(92.3)	<0.001
Freezing kills bacteria causing food-borne illnesses	158 (33.9)	103(27.9)	14(43.8)	41(63.1)	<0.001
Contaminated fish always changes colour, odour or taste	14 (3.0)	11(3.0)	0(0.0)	3(4.6)	0.456
Fresh fish products should be stored below 4^0^C	177 (38.0)	135(36.6)	3(9.4)	39(60.0)	<0.001

∗ col% = Column percentage.

**Table 3a:** Attitude towards food safety requirements among fish handlers in the fresh Nile perch Market value chain in Uganda

Indicator	Export market chain n = 252; Freq(arow%)					Local market chain n = 214; Freq(row%)					X^2^ P-value
	Strongly Agree	Agree	No idea	Disagree	Strongly disagree	Strongly Agree	Agree	No idea	Disagree	Strongly disagree	
One of the most important responsibilities of my job is to handle the fish in a way that keeps it safe for consumption.	203(80.6)	46(18.3)	0 (0.0)	3(1.2)	0 (0.0)	99(46.3)	102(47.7)	0 (0.0)	13(6.1)	0 (0.0)	<0.001
Learning more about food hygiene is important for my job	192(76.2)	51(20.2)	8(3.2)	1(0.4)	0 (0.0)	116(54.2)	88(41.1)	3(1.4)	7(3.3)	0 (0.0)	<0.001
The price and taste of fish are more important than food safety	30(11.9)	22(8.7)	15(6.0)	125(49.6)	60(23.8)	29(13.6)	32(15.0)	12(5.6)	114(53.3)	27(12.6)	0.016
Foodborne illnesses can have a negative effect on the health and economic status of society.	135(53.6)	98(38.9)	16(6.3)	3(1.2)	0 (0.0)	92(43.0)	84(39.3)	35(16.4)	3(1.4)	0 (0.0)	0.004
Written food safety policies, procedures, and well-kept records are necessary to keep food safe	132(52.4)	45(17.9)	68(27.0)	7(2.8)	0(0.0)	45(21.0)	16(7.5)	145(67.8)	5(2.3)	3(1.4)	<0.001
It is important to have regular food safety inspections of our fish handling facilities	193(76.6)	58(23.0)	0(0.0)	1(0.4)	0 (0.0)	129(60.3)	83(38.8)	1(0.5)	1(0.5)	0 (0.0)	0.002
Training in sanitation practices does not apply to practical everyday life	43(17.1)	37(14.7)	6(2.4)	111(44.0)	55(21.8)	13(6.1)	14(6.5)	2(0.9)	146(68.2)	39(18.2)	<0.001
If knives and cutting boards are not properly cleaned, the fish products handled could make people sick.	188(74.6)	59(23.4)	3(1.2)	1(0.4)	1(0.4)	98(45.8)	97(45.3)	5(2.3)	12(5.6)	2(0.9)	<0.001
Some contact surfaces could contaminate fish with foodborne pathogens	167(66.5)	81(32.3)	1(0.4)	2(0.8)	0 (0.0)	113(52.8)	97(45.3)	3(1.4)	1(0.5)	0 (0.0)	0.016
Maintaining a clean working environment is a good way to control food safety	200(79.7)	51(20.3)	0 (0.0)	0 (0.0)	0 (0.0)	125(58.4)	89(41.6)	0 (0.0)	0 (0.0)	0 (0.0)	<0.001
Health status of the workers should be evaluated before employment	169(67.1)	77(30.6)	0(0.0)	6(2.4)	0(0.0)	106(49.5)	103(48.1)	1(0.5)	3(1.4)	1(0.5)	0.001
Fish handlers suffering from foodborne diseases should not be allowed to go to work and should stay away from the fish handling areas where they work.	198(78.6)	51(20.2)	1(0.4)	2(0.8)	0 (0.0)	64(29.9)	140(65.4)	4(1.9)	6(2.8)	0 (0.0)	<0.001
Fish handlers who have abrasions or cuts on hands should not touch fish without correctly covering their cuts possibly with gloves	205(81.3)	46(18.3)	0 (0.0)	1(0.4)	0 (0.0)	138(64.5)	73(34.1)	0 (0.0)	3(1.4)	0 (0.0)	<0.001
Personal cleanliness is highly important when we are at work	189(75.0)	61(24.2)	2(0.8)	0(0.0)	0 (0.0)	110(51.4)	97(45.3)	1(0.5)	6(2.8)	0 (0.0)	<0.001
If fish handlers do not wash their hands after every time they handle anything other than fish, their fish will make people sick	136(54.0)	97(38.5)	6(2.4)	13(5.2)	0(0.0)	44(20.6)	42(19.6)	19(8.9)	92(43.0)	17(7.9)	<0.001
Wearing masks is an important practice to reduce the risk of food contamination	191(75.8)	57(22.6)	0(0.0)	1(0.4)	3(1.2)	89(41.6)	111(51.9)	1(0.5)	11(5.1)	2(0.9)	<0.001
Wearing gloves is an important practice to reduce the risk of food contamination.	170(67.5)	71(28.2)	0(0.0)	8(3.2)	3(1.2)	77(36.0)	65(30.4)	1(0.5)	66(30.8)	5(2.3)	<0.001
Wearing caps is an important practice to reduce the risk of food contamination.	196(77.8)	47(18.7)	0 (0.0)	8(3.2)	1(0.4)	75(35.0)	79(36.9)	0 (0.0)	50(23.4)	10(4.7)	<0.001

a row% = Row percentage.

**Table 3b:** Attitude towards food safety requirements among fish handlers in the fresh Nile perch segment of the value chain in Uganda

Indicator	Landing site n = 369; Freq(∗row%)					Market stalls n = 32; Freq(row%)					Factory n = 65; Freq(row%)					
	S Agree	Agree	No idea	Disagree	S disagree	S Agree	Agree	No idea	Disagree	S disagree	S Agree	Agree	No idea	Disagree	S disagree	X^2^ P-value
One of the most important responsibilities of my job is to handle the fish in a way that keeps it safe for consumption.	233(63.1)	124(33.6)	0 (0.0)	12(3.3)	0 (0.0)	14(43.8)	14(43.8)	0 (0.0)	4(12.5)	0 (0.0)	55(84.6)	10(15.4)	0 (0.0)	0(0.0)	0 (0.0)	<0.001
Learning more about food hygiene is important for my job	241(65.3)	111(30.1)	11(3.0)	6(1.6)	0 (0.0)	20(62.5)	10(31.3)	0(0.0)	2(6.3)	0 (0.0)	47(72.3)	18(27.7)	0(0.0)	0(0.0)	0 (0.0)	0.206
The price and taste of fish are more important than food safety	52(14.1)	48(13.0)	17(4.6)	198(53.7)	54(14.6)	4(12.5)	3(9.4)	6(18.8)	14(43.8)	5(15.6)	3(4.6)	3(4.6)	4(6.2)	27(41.5)	28(43.1)	<0.001
Foodborne illnesses can have a negative effect on the health and economic status of society.	185(50.1)	137(37.1)	44(11.9)	3(0.8)	0 (0.0)	12(37.5)	16(50.0)	4(12.5)	0(0.0)	0 (0.0)	30(46.2)	29(44.6)	3(4.6)	3(4.6)	0 (0.0)	0.055
Written food safety policies, procedures, and well-kept records are necessary to keep food safe	140(37.9)	32(8.7)	189(51.2)	7(1.9)	1(0.3)	7(21.9)	4(12.5)	17(53.1)	2(6.3)	2(6.3)	30(46.2)	25(38.5)	7(10.8)	3(4.6)	0(0.0)	<0.001
It is important to have regular food safety inspections of our fish handling facilities	265(71.8)	101(27.4)	1(0.3)	2(0.5)	0 (0.0)	13(40.6)	19(59.4)	0(0.0)	0(0.0)	0 (0.0)	44(67.7)	21(32.3)	0(0.0)	0(0.0)	0 (0.0)	0.02
Training in sanitation practices does not apply to practical everyday life	51(13.8)	44(11.9)	6(1.6)	207(56.1)	61(16.5)	2(6.3)	0(0.0)	0(0.0)	17(53.1)	13(40.6)	3(4.6)	7(10.8)	2(3.1)	33(50.8)	20(30.8)	0.003
If knives and cutting boards are not properly cleaned, the fish products handled could make people sick.	236(64.0)	113(30.6)	5(1.4)	13(3.5)	2(0.5)	17(53.1)	13(40.6)	2(6.3)	0(0.0)	0(0.0)	33(50.8)	30(46.2)	1(1.5)	0(0.0)	1(1.5)	0.058
Some contact surfaces could contaminate fish with foodborne pathogens	225(61.0)	139(37.7)	4(1.1)	1(0.3)	0 (0.0)	18(56.3)	14(43.8)	0(0.0)	0(0.0)	0 (0.0)	37(57.8)	25(39.1)	0(0.0)	2(3.1)	0 (0.0)	0.196
Maintaining a clean working environment is a good way to control food safety	252(68.5)	116(31.5)	0 (0.0)	0 (0.0)	0 (0.0)	20(62.5)	12(37.5)	0 (0.0)	0 (0.0)	0 (0.0)	53(81.5)	12(18.5)	0 (0.0)	0 (0.0)	0 (0.0)	0.068
Health status of the workers should be evaluated before employment	220(59.6)	141(38.2)	1(0.3)	6(1.6)	1(0.3)	20(62.5)	12(37.5)	0(0.0)	0(0.0)	0(0.0)	35(53.8)	27(41.5)	0(0.0)	3(4.6)	0(0.0)	0.829
Fish handlers suffering from foodborne diseases should not be allowed to go to work and should stay away from the fish handling areas where they work.	205(55.6)	152(41.2)	5(1.4)	7(1.9)	0 (0.0)	16(50.0)	16(50.0)	0(0.0)	0(0.0)	0 (0.0)	41(63.1)	23(35.4)	0(0.0)	1(1.5)	0 (0.0)	0.694
Fish handlers who have abrasions or cuts on hands should not touch fish without correctly covering their cuts possibly with gloves	281(76.2)	84(22.8)	0 (0.0)	4(1.1)	0 (0.0)	16(50.0)	16(50.0)	0 (0.0)	0(0.0)	0 (0.0)	46(70.8)	19(29.2)	0 (0.0)	0(0.0)	0 (0.0)	0.012
Personal cleanliness is highly important when we are at work	236(64.0)	124(33.6)	3(0.8)	6(1.6)	0 (0.0)	14(43.8)	18(56.3)	0(0.0)	0(0.0)	0 (0.0)	49(75.4)	16(24.6)	0(0.0)	0(0.0)	0 (0.0)	0.06
If fish handlers do not wash their hands after every time they handle anything other than fish, their fish will make people sick	144(39.0)	99(26.8)	21(5.7)	92(24.9)	13(3.5)	11(34.4)	9(28.1)	2(6.3)	6(18.8)	4(12.5)	25(38.5)	31(47.7)	2(3.1)	7(10.8)	0(0.0)	0.003
Wearing masks is an important practice to reduce the risk of food contamination	220(59.6)	135(36.6)	1(0.3)	10(2.7)	3(0.8)	13(40.6)	15(46.9)	0(0.0)	2(6.3)	2(6.3)	47(72.3)	18(27.7)	0(0.0)	0(0.0)	0(0.0)	0.018
Wearing gloves is an important practice to reduce the risk of food contamination.	182(49.3)	109(29.5)	1(0.3)	69(18.7)	8(2.2)	21(65.6)	7(21.9)	0(0.0)	4(12.5)	0(0.0)	44(67.7)	20(30.8)	0(0.0)	1(1.5)	0(0.0)	0.021
Wearing caps is an important practice to reduce the risk of food contamination.	207(56.1)	97(26.3)	0 (0.0)	54(14.6)	11(3.0)	17(53.1)	11(34.4)	0 (0.0)	4(12.5)	0(0.0)	47(72.3)	18(27.7)	0 (0.0)	0(0.0)	0(0.0)	0.015

∗ row% = Row percentage.

**Table 4a:** Food safety practices for the fish handlers within the fresh Nile perch Market value chain in Uganda

Indicator	Overall	Market chain of interest		
		Export	Local	
	Freq. n = 466 (percentage)	Freq. n = 252 (acol%)	Freq. n = 214 (col%)	X^2^ P-value
**Source of water for cleaning**				<0.001
municipal water	294(63.1)	229(90.9)	65(30.4)	
rain water	5(1.1)	0(0.0)	5(2.3)	
lake water	159(34.1)	15(6.0)	144(67.3)	
others	8(1.7)	8(3.2)	0(0.0)	
**What is used to wash hands**				<0.001
water and soap	206(44.2)	117(46.4)	89(41.6)	
soap, water and sanitizer	131(28.1)	125(49.6)	6(2.8)	
water only	129(27.7)	10(4.0)	119(55.6)	
**How often are hands washed**				<0.001
once told	1(0.2)	0(0.0)	1(0.5)	
before touching fish	281(60.4)	216(85.7)	65(30.5)	
at start and end of work	164(35.3)	32(12.7)	132(62.0)	
others	19(4.1)	4(1.6)	15(7.0)	
**Where are meals consumed**				<0.001
nearby restaurant	295(63.6)	161(63.9)	134(63.2)	
staff canteen	103(22.2)	89(35.3)	14(6.6)	
work place	66(14.2)	2(0.8)	64(30.2)	
**Finger nails appearance**				<0.001
neat, long and painted	22(4.7)	3(1.2)	19(8.9)	
neat and short	407(87.3)	248(98.4)	159(74.3)	
neat short and painted	9(1.9)	0(0.0)	9(4.2)	
others	28(6)	1(0.4)	27(12.6)	
**Sickness and wounds or cuts**				<0.001
get treatment and go to work	178(38.2)	32(12.7)	146(68.2)	
stay away from work until ok	248(53.2)	217(86.1)	31(14.5)	
go to work	37(7.9)	0(0.0)	37(17.3)	
others	3(0.6)	3(1.2)	0(0.0)	
**Frequency of medical examination**				<0.001
once in six months	347(74.5)	244(96.8)	103(48.1)	
once a year	60(12.9)	0(0.0)	60(28.0)	
never	50(10.7)	5(2.0)	45(21.0)	
others	9(1.9)	3(1.2)	6(2.8)	
**Cleaning equipment**				<0.001
soap and water	270(57.9)	205(81.3)	65(30.4)	
water only	156(33.5)	11(4.4)	145(67.8)	
soap, water and sanitizer	36(7.7)	32(12.7)	4(1.9)	
others	4(0.9)	4(1.6)	0(0.0)	
**When is in-working cleaning done**				<0.001
after every batch	241(51.7)	191(75.8)	50(23.4)	
once a week	3(0.6)	0(0.0)	3(1.4)	
once a day	207(44.4)	51(20.2)	156(72.9)	
others	15(3.2)	10(4.0)	5(2.3)	
**How is fish kept**				<0.001
freezer, cold room, refrigerator	244(52.4)	208(82.5)	36(16.8)	
in open with ice	86(18.5)	24(9.5)	62(29.0)	
in open without ice	90(19.3)	3(1.2)	87(40.7)	
others	46(9.9)	17(6.7)	29(13.6)	
**How long is fish kept**				<0.001
less than 6hrs	96(20.6)	74(29.4)	22(10.3)	
6–12hrs	98(21)	26(10.3)	72(33.6)	
12–24hrs	203(43.6)	92(36.5)	111(51.9)	
more than 24hrs	69(14.8)	60(23.8)	9(4.2)	
**Frequency of fish quality checks**				0.001
per batch	430(92.3)	229(90.9)	201(93.9)	
daily	30(6.4)	23(9.1)	7(3.3)	
others	6(1.3)	0(0.0)	6(2.8)	
**Frequency of regulatory facility inspection**				<0.001
monthly	148(31.8)	84(33.5)	64(29.9)	
quarterly	240(51.6)	145(57.8)	95(44.4)	
annually	48(10.3)	13(5.2)	35(16.4)	
others	29(6)	9(3.6)	20(9.3)	

a col% = Column percentage.

**Table 4b:** Food safety practices for the fish handlers within the fresh Nile perch Segment of the value chain in Uganda

Indicator	Overall	segment of the value chain			
		Landing site	Market stalls	Factory	
	Freq. n = 466 (percentage)	Freq. n = 369 (∗col%)	Freq. n= (col%)	Freq. n = 65 (col%)	X^2^ P-value
**Source of water for cleaning**					<0.001
municipal water	294(63.1)	208(56.4)	31(96.9)	55(84.6)	
rain water	5(1.1)	4(1.1)	1(3.1)	0(0.0)	
lake water	159(34.1)	153(41.5)	0(0.0)	6(9.2)	
others	8(1.7)	4(1.1)	0(0.0)	4(6.2)	
**What is used to wash hands**					<0.001
water and soap	206(44.2)	177(48.0)	25(78.1)	4(6.2)	
soap, water and sanitizer	131(28.1)	70(19.0)	0(0.0)	61(93.8)	
water only	129(27.7)	122(33.1)	7(21.9)	0(0.0)	
**How often are hands washed**					<0.001
once told	1(0.2)	1(0.3)	0(0.0)	0(0.0)	
before touching fish	281(60.4)	206(56.0)	18(56.3)	57(87.7)	
at start and end of work	164(35.3)	146(39.7)	14(43.8)	4(6.2)	
others	19(4.1)	15(4.1)	0(0.0)	4(6.2)	
**Where are meals consumed**					<0.001
nearby restaurant	295(63.6)	274(74.3)	20(66.7)	1(1.5)	
staff canteen	103(22.2)	38(10.3)	2(6.7)	63(96.9)	
work place	66(14.2)	57(15.4)	8(26.7)	1(1.5)	
**Finger nails appearance**					0.004
neat, long and painted	22(4.7)	22(6.0)	0(0.0)	0(0.0)	
neat and short	407(87.3)	312(84.6)	30(93.8)	65(100.0)	
neat short and painted	9(1.9)	7(1.9)	2(6.3)	0(0.0)	
others	28(6)	28(7.6)	0(0.0)	0(0.0)	
**Sickness and wounds or cuts**					<0.001
get treatment and go to work	178(38.2)	152(41.2)	24(75.0)	2(3.1)	
stay away from work until ok	248(53.2)	179(48.5)	6(18.8)	63(96.9)	
go to work	37(7.9)	35(9.5)	2(6.3)	0(0.0)	
others	3(0.6)	3(0.8)	0(0.0)	0(0.0)	
**Frequency of medical examination**					<0.001
once in six months	347(74.5)	258(69.9)	27(84.4)	62(95.4)	
once a year	60(12.9)	59(16.0)	1(3.1)	0(0.0)	
never	50(10.7)	47(12.7)	0(0.0)	3(4.6)	
others	9(1.9)	5(1.4)	4(12.5)	0(0.0)	
**Cleaning equipment**					<0.001
soap and water	270(57.9)	213(57.7)	19(59.4)	38(58.5)	
water only	156(33.5)	142(38.5)	13(40.6)	1(1.5)	
soap, water and sanitizer	36(7.7)	11(3.0)	0(0.0)	25(38.5)	
others	4(0.9)	3(0.8)	0(0.0)	1(1.5)	
**When is in-working cleaning done**					<0.001
after every batch	241(51.7)	177(48.0)	12(37.5)	52(80.0)	
once a week	3(0.6)	3(0.8)	0(0.0)	0(0.0)	
once a day	207(44.4)	183(49.6)	17(53.1)	7(10.8)	
others	15(3.2)	6(1.6)	3(9.4)	6(9.2)	
**How is fish kept**					<0.001
freezer, cold room, refrigerator	244(52.4)	169(45.8)	14(43.8)	61(93.8)	
in open with ice	86(18.5)	80(21.7)	2(6.3)	4(6.2)	
in open without ice	90(19.3)	78(21.1)	12(37.5)	0(0.0)	
others	46(9.9)	42(11.4)	4(12.5)	0(0.0)	
**How long is fish kept**					<0.001
less than 6hrs	96(20.6)	65(17.6)	3(9.4)	28(43.1)	
6–12hrs	98(21)	77(20.9)	16(50.0)	5(7.7)	
12–24hrs	203(43.6)	173(46.9)	9(28.1)	21(32.3)	
more than 24hrs	69(14.8)	54(14.6)	4(12.5)	11(16.9)	
**Frequency of fish quality checks**					0.669
per batch	430(92.3)	339(91.9)	31(96.9)	60(92.3)	
daily	30(6.4)	24(6.5)	1(3.1)	5(7.7)	
others	6(1.3)	6(1.6)	0(0.0)	0(0.0)	
**Frequency of regulatory facility inspection**					<0.001
monthly	148(31.8)	125(33.9)	9(28.1)	14(21.9)	
quarterly	240(51.6)	173(46.9)	17(53.1)	50(78.1)	
annually	48(10.3)	46(12.5)	2(6.3)	0(0.0)	
others	29(6)	25(6.8)	4(12.5)	0(0.0)	

∗ col% = Column percentage.

**Table 5:** Summary analysis of the different components of knowledge and attitude of fish handlers

	Market chain of interest				Segment of the value chain			
	Overall	Export	Local	ꭓ^2^2 P-value	Landing site	Market stalls	Factory	ꭓ^2^ P-value
	Freq (acol%) n = 466	Freq (col%) n = 252	Freq (col%) n = 214		Freq (col%) n = 369	Freq (col%) n = 32	Freq (col%) n = 65	
**Knowledge**				**<0.001**				**<0.001**
Good knowledge	232 (49.8)	188 (74.6)	44 (20.6)		168 (45.5)	6 (18.8)	58 (89.2)	
Poor knowledge	234 (50.2)	64 (25.4)	170(79.4)		201 (54.5)	26 (81.3)	7 (10.8)	
**Attitude**				**<0.001**				**<0.001**
Good attitude	128 (27.5)	107 (42.5)	21 (9.8)		92 (24.9)	4 (12.5)	32 (49.2)	
Fair attitude	182 (39.1)	112 (44.4)	70 (32.7)		144 (39.0)	14 (43.8)	24 (36.9)	
Poor attitude	156 (33.5)	33 (13.1)	123(57.5)		133 (36.0)	14 (43.8)	9 (13.8)	

a col% = Column percentage.

**Table 6:** Association of market chain and knowledge of food safety requirements

Characteristics	Bivariate analysis	Multivariable analysis
	β Coeff^a^ (95 % CI) P-value	β Coeff (95 % CI) P-value
Market Chain		
Export	Ref^b^	Ref
Local	−2.63(-3.02, −2.24) <0.001	−1.87(-2.35, −1.39) <0.001
**Gender of respondent**		
Female	Ref	Ref
Male	−0.03(-0.69, 0.64) 0.939	0.81(0.26, 1.36) 0.004
**Age category**		
18–20yrs	Ref	Ref
20–30yrs	1.50(-0.16, 3.17) 0.077	0.74(-0.55, 2.02) 0.261
30–40yrs	1.85(0.18, 3.51) 0.030	0.81(-0.50, 2.12) 0.225
Above 40yrs	2.05(0.34, 3.76) 0.019	1.00(-0.42, 2.42) 0.167
**Highest Education level attained**		
No formal education	Ref	Ref
Primary	0.42(-0.29, 1.14) 0.247	−0.18(-0.84, 0.48) 0.589
Secondary	1.64(0.90, 2.37) <0.001	0.16(-0.56, 0.88) 0.663
Tertiary	4.07(3.26, 4.88) <0.001	1.64(0.70, 2.58) 0.001
**Average monthly income**		
<300,000	Ref	Ref
300,000–600,000	0.89(0.42, 1.36) <0.001	0.72(0.26, 1.17) 0.002
600,001–900,000	2.61(1.83, 3.39) <0.001	0.76(-0.04, 1.56) 0.063
>900,000	3.03(1.97, 4.09) <0.001	0.69(-0.34, 1.72) 0.187
**Years of business experience**		
5yrs or less	Ref	Ref
6–10yrs	0.34(-0.19, 0.87) 0.212	0.41(-0.06, 0.88) 0.086
11–15yrs	0.24(-0.55, 1.03) 0.546	−0.85(-1.55, −0.16) 0.017
>15yrs	0.77(0.10, 1.44) 0.024	−0.32(-1.01, 0.38) 0.373
**Role of Respondent**		
Worker	Ref	Ref
Owner	−0.72(-1.22, −0.21) 0.006	0.35(-0.10, 0.79) 0.129
**Had any Food Safety training**		
No	Ref	Ref
Yes	2.38(1.98, 2.79) <0.001	0.97(0.53, 1.40) <0.001

a Coefficient.

b Reference variable.

**Table 7:** Association of market chain and attitude of fish handlers towards food safety requirements

**Characteristics**	Attitude	
	**Bivariate analysis**	**Multivariable analysis**
	**β Coeff(95 % CI) P-value**	**β Coeff(95 % CI) P-value**
**Market Chain**		
Export	Ref	Ref
Local	−7.79(-8.96, −6.62) <0.001	−5.80(-7.36, −4.24) <0.001
**Gender of respondent**		
Female	Ref	Ref
Male	2.99(1.01, 4.97) 0.003	3.43(1.65, 5.21) <0.001
**Age category**		
18–20yrs	Ref	Ref
20–30yrs	0.22(-4.82, 5.27) 0.931	0.15(-4.02, 4.33) 0.942
30–40yrs	0.61(-4.43, 5.66) 0.812	0.95(-3.32, 5.22) 0.662
Above 40yrs	0.61(-4.57, 5.80) 0.816	0.81(-3.81, 5.43) 0.732
**Highest Education level attained**		
No formal educ	Ref	Ref
Primary	5.25(3.01, 7.49) <0.001	3.19(1.05, 5.33) 0.004
Secondary	9.98(7.68, 12.28) <0.001	5.88(3.52, 8.23) <0.001
Tertiary	11.91(9.37, 14.45) <0.001	6.09(3.03, 9.15) <0.001
**Average monthly income**		
<300,000	Ref	Ref
300,000–600,000	−0.21(-1.69, 1.27) 0.778	−0.13(-1.59, 1.34) 0.864
600,001–900,000	4.91(2.46, 7.35) <0.001	2.31(-0.30, 4.92) 0.083
>900,000	5.28(1.94, 8.62) 0.002	1.23(-2.11, 4.57) 0.470
**Years of business experience**		
5yrs or less	Ref	Ref
6–10yrs	−1.54(-3.13, 0.05) 0.058	−0.27(-1.80, 1.26) 0.728
11–15yrs	−0.75(-3.12, 1.62) 0.537	−1.88(-4.15, 0.39) 0.105
>15yrs	1.24(-0.76, 3.25) 0.224	−1.27(-3.53, 1.00) 0.273
**Role of Respondent**		
Worker	Ref	Ref
Owner	−1.93(-3.45, −0.40) 0.013	1.34(-0.12, 2.81) 0.072
**Had any Food Safety training**		
No	Ref	Ref
Yes	4.75(3.43, 6.06) <0.001	0.78(-0.64, 2.19) 0.280
