# Supplementary material for: Knowledge, attitudes and practices of fresh Nile perch value chain handlers towards food safety requirements in Uganda
Source: Heliyon. 2024 May 20;10(10):e31432. doi: 10.1016/j.heliyon.2024.e31432 (PMC11141374; doi:10.1016/j.heliyon.2024.e31432)
Supplement: Multimedia component 1 [file mmc1.docx]

**Food safety knowledge, attitudes and practices of key actors in the fresh Nile perch value chain in Uganda**

| Serial No: ________________________  **Introduction**  Dear Participant  My Name is ______________________, a researcher from Makerere University College of Agricultural and Environmental Sciences. I am conducting a study titled ***“Food Safety Aspects in the Fresh Nile perch Value Chain in Uganda.”*** You have been selected on merit as a vital source of information towards the success of the study. Please note that any information that you will provide will be used mainly for academic purposes and will be treated with utmost confidentiality. Your willingness to participate in this exercise is highly appreciated. | | |
| --- | --- | --- |
| **Section A: Respondent’s Socio-demographic characteristic** | | |
| *1.* | Gender of the respondent. | 1. Male 2. Female |
| *2.* | How old are you? (In complete years). | 1. 18- 20 years 2. 20-30 years 3. 30-40 years 4. Above 40 years ________________________ |
| *3.* | What is your highest level of education? | 1. No formal education  2. Primary  3. Secondary  4. Tertiary |
| *4.* | Average monthly income in Uganda shillings | 1. < 300,000 2. 300,000-600,000 3. 600,000 -900,000 4. >900,000 |
| *5..* | What exactly do you do in the Nile perch value chain? | 1. Fisherman  2. Transporter  3. Handler  4. Supervisor/ Manager  5. Trader |
| *6.* | Years of business experience | 1. ≤ 5years 2. 6-10 years 3. 11- 15 years 4. > 15 years |
| *7* | Role of respondent | 1. Worker 2. Owner |
| *8.* | Have you ever had any food safety training? If yes, please provide additional information on what training, by who, when and how often… | 1. Yes 2. No |

| **Section B: Food safety knowledge of fish handlers** |
| --- |

| **#** | **Statement**  ***Note: Please tick what you know as the correct answer*** | 1. **True** | 1. **False** | 1. **I don’t know** |
| --- | --- | --- | --- | --- |
| 1 | Washing hands before handling fish, using only water reduces the risk of microbial contamination of fish. |  |  |  |
| 2 | Using gloves while handling fish reduces the risk of food contamination |  |  |  |
| 3 | Eating and drinking from the work place increases the risk of food contamination |  |  |  |
| 4 | Proper cleaning and handling of fish contact surfaces reduces the risk of food contamination |  |  |  |
| 5 | Washing equipment without soap leaves them free of contamination. |  |  |  |
| 6 | Children, healthy adults, pregnant women and older individuals are at equal risk for food poisoning |  |  |  |
| 7 | Diseases like HIV, COVID-19 and Ebola can be transmitted by eating contaminated fish |  |  |  |
| 8 | Diseases like diarrhea, dysentery, typhoid, cholera etc. can be transmitted by eating contaminated fish |  |  |  |
| 9 | E.coli, Salmonella, Staphylococcus are among the food borne pathogens |  |  |  |
| 10 | Food borne pathogens are naturally existent in the environment (on people, working surfaces, animals, and sometimes even on food itself) |  |  |  |
| 11 | Some food borne pathogens can naturally exist in water; and water in some cases may contaminate the fishery products. |  |  |  |
| 12 | Cross contamination is when microorganisms from a contaminated item/person are transferred (by the fish handler, animal, processing aid, or equipment) to fish/food. |  |  |  |
| 13 | Freezing kills all the bacteria that may cause food-borne illness |  |  |  |
| 14 | Contaminated fish and fishery products always have some change in color, odor or taste. |  |  |  |
| 15 | The correct temperature for storing fresh fish and fishery products is below 4^0^C |  |  |  |

| **Section C: Food safety attitudes of fish handlers** |
| --- |

| **#** | **Statement**  ***Note: Please tick the column that best represents your belief*** | 1. **Strongly Agree** | 1. **Agree** | 1. **No Idea** | 1. **Disagree** | 1. **Strongly Disagree** |
| --- | --- | --- | --- | --- | --- | --- |
| 1 | One of the most important responsibilities of my job is to handle the fish in a way that keeps it safe for consumption. |  |  |  |  |  |
| 2 | Learning more about Food hygiene is important for my job. |  |  |  |  |  |
| 3 | The price and taste of fish are more important than food safety |  |  |  |  |  |
| 4 | Foodborne illnesses can have a negative effect on the health and economic status of society. |  |  |  |  |  |
| 5 | Written food safety policies, procedures, and well-kept records are necessary to keep food safe. |  |  |  |  |  |
| 6 | It is important to have regular food safety inspections of our fish handling facilities |  |  |  |  |  |
| 7 | Training in sanitation practices does not apply to practical everyday life |  |  |  |  |  |
| 8 | If knives and cutting boards are not properly cleaned, the fish products handled could make people sick. |  |  |  |  |  |
| 9 | Some contact surfaces could contaminate fish with foodborne pathogens |  |  |  |  |  |
| 10 | Maintaining a clean working environment is a good way to control food safety |  |  |  |  |  |
| 11 | Health status of the workers should be evaluated before employment |  |  |  |  |  |
| 12 | Fish handlers suffering from foodborne diseases should not be allowed to go to work and should stay away from the fish handling areas where they work. |  |  |  |  |  |
| 13 | Fish handlers who have abrasions or cuts on hands should not touch fish without correctly covering their cuts possibly with gloves. |  |  |  |  |  |
| 14 | Personal cleanliness is highly important when we are at work |  |  |  |  |  |
| 15 | If fish handlers do not wash their hands after every time they handle anything other than fish, their fish will make people sick. |  |  |  |  |  |
| 16 | Wearing masks is an important practice to reduce the risk of food contamination. |  |  |  |  |  |
| 17 | Wearing gloves is an important practice to reduce the risk of food contamination. |  |  |  |  |  |
| 18 | Wearing caps is an important practice to reduce the risk of food contamination. |  |  |  |  |  |

| **Section C: Food safety practices of fish handlers** |
| --- |

| **#** | ***Note: Tick the most appropriate answer. If you choose others, please provide more information*** |
| --- | --- |
| 1 | What is the source of water used for cleaning purposes at your facility?   1. Municipal piped water 2. Rain water 3. Lake water 4. Others specify…….. |
| 2 | What do you always use to wash your hands?   1. Water and soap 2. Soap, water and sanitizer 3. Water only 4. Others specify…….. |
| 3 | How often do you wash your hands?   1. Once told by supervisor or customer 2. Every time before touching fish 3. At start and end of work 4. Others specify…….. |
| 4 | Where do you normally have meals (eat or drink) from while at your work?   1. Nearby restaurant 2. Staff canteen 3. Work space 4. Others specify…….. |
| 5 | How do you maintain fingernails?   1. Neat, long, and painted 2. Neat and short, 3. Neat, short, and painted 4. Others specify…….. |
| 6 | What do you do when you are sick with flu, diarrhea, skin rush, and or cuts/ wounds?   1. Get treatment and go to work 2. Stay away from work until ok 3. Go to work 4. Others specify…….. |
| 7 | How often do the municipality health officials medically examine you for fitness to handle fish/food products?   1. Once in 6 months 2. Once a year 3. Never 4. Others |
| 8 | What do you always use to clean equipment?   1. Soap and water 2. Water only 3. Soap, water and sanitizer 4. Others specify…….. |
| 9 | When do you normally clean the working area and equipment?   1. After every batch 2. Once a week 3. Once a day 4. Others specify…….. |
| 10 | How do you keep the fish?   1. In a freezer or cold room, refrigerator 2. In the open with ice 3. In the open without ice 4. Others specify…….. |
| 11 | How long do you keep the fish under the above storage conditions before selling or disposing off?   1. Less than 6 hours 2. 6-12 hours 3. 12-24 hours 4. Others specify…….. |
| 12 | How often do you perform the fish quality checks?   1. Per batch 2. Daily 3. Weekly 4. Others specify…… |
| 13 | How often is your facility inspected by the authorities?   1. Monthly 2. Quarterly 3. Annually 4. Others specify |
