# Supplementary material for: Knowledge, attitudes and practices of fresh Nile perch value chain handlers towards food safety requirements in Uganda
Source: Heliyon. 2024 May 20;10(10):e31432. doi: 10.1016/j.heliyon.2024.e31432 (PMC11141374; doi:10.1016/j.heliyon.2024.e31432)
Supplement: Multimedia component 2 [file mmc2.docx]

**Supplementary Tables**

Table ST1. Registered and Operational Fish Landing Sites on Lake Victoria

| **District Landing site No. of fish handlers** | | |
| --- | --- | --- |
| **Zone 1; Lake Victoria South Western Region** | | |
| Masaka | Kachanga 6  Ddimo 7 |  |
| Rakai | Kasensero 27 |  |
| **Zone 2; Lake Victoria Kalangala Islands** | | |
| Kalangala | Ttubi 23  Kyagalanyi 23  Nakatiba 22 |  |
| **Zone 3; Lake Victoria Central** | | |
| Wakiso | Kasenyi 19  Kigungu 17  Greenfields 5  Fresh Perch Bugonga 6 |  |
| **Zone 4; Lake Victoria Eastern Region A** | | |
| Buikwe | Kiyindi 50  Ssenyi 50 |  |
| Mukono | Katosi 70 |  |
| **Zone 5; Lake Victoria Eastern Region B** | | |
| Jinja | Fresh Perch 3 |  |
| Mayuge | Bwondha 41 |  |
| **Total 369** | |  |

Table ST2. List of Licensed and Operational Fish Markets around Lake Victoria Basin

| **S/N** | **Market** | **Location** | **No. of fish handlers** |
| --- | --- | --- | --- |
| **1** | Malaba fish market stalls | Tororo District | 4 |
| **2** | Nakawa fish market stalls | Nakawa Division- Kampala District | 2 |
| **3** | Busia fish market stall | Busia District | 4 |
| **4** | Jinja fish market stalls | Jinja District | 3 |
| **5** | Mukono fish market stalls | Mukono District | 1 |
| **6** | Kalerwe fish market stalls | Kawempe Division- Kampala District | 5 |
| **7** | Nyendo fish market stalls | Masaka District | 3 |
| **8** | Ggaba Fish Market | Makindye Division- Kampala District | 5 |
| **9** | Busega Fish market | Rubaga Division – Kampala District | 5 |
|  | **Total** |  | **32** |

Table ST3. List of Licensed and Operational Fish Processing Factories

| **S/N** | **Name of Fish Factory** | **Location** | **No. of fish handlers** |
| --- | --- | --- | --- |
| **1** | Green Fields (U) Limited | Entebbe, Wakiso | 5 |
| **2** | Fresh Perch Limited | Entebbe, Wakiso | 6 |
| **2** | Karmic Foods Limited | Entebbe, Wakiso | 6 |
| **4** | Lake Perch Limited | Katabi, Wakiso | 3 |
| **5** | Iftra Uganda Limited | Kanyanya, Kampala | 5 |
| **6** | Sese Fresh Packers | Rubaga, Kampala | 2 |
| **7** | Ngege Limited | Luzira, Kampala | 6 |
| **8** | Lake Bounty Limited | Ntinda, Kampala | 5 |
| **9** | Byansi Fisheries Company Limited | Kalisizo, Masaka | 3 |
| **10** | Mpongo Limited | Luzira, Kampala | 4 |
| **11** | Nyanza Perch Limited | Jinja city, Jinja | 5 |
| **12** | Fresh Perch Jinja | Jinja city, Jinja | 4 |
| **13** | Victoria Treasures Limited | Garuga, Wakiso | 3 |
| **14** | Ask International Limited | Lugala, Kampala | 2 |
| **15** | Tropical Fish Company | Kawuku, Wakiso | 6 |
|  | **Total** |  | **65** |
